# A Critical Review of Surveys Emphasizing on Routing in Wireless Sensor Networks—An Anatomization under General Survey Design Framework

**DOI:** 10.3390/s17081713

**Published:** 2017-07-26

**Authors:** Waqas Rehan, Stefan Fischer, Maaz Rehan

**Affiliations:** 1 Institute of Telematics (ITM), University of Luebeck, Ratzeburger Allee 160, 23562 Luebeck, Germany; fischer@itm.uni-luebeck.de; 2 COMSATS Institue of Information Technology (CIIT), Quaid Avenue, 47040 Wah Cantt, Pakistan; maazrehan@ciitwah.edu.pk

**Keywords:** general survey design framework, big data analysis & issues, WSNs, hierarchical, energy-efficient, multipath, computationally-intelligent, secure, QoS-based and geographic

## Abstract

A large number of routing-related surveys are published so far for Wireless Sensor Networks (WSNs) that exhibit either complete or partial emphasis on routing in WSNs. These surveys classify and discuss the relevant routing protocols published mainly in the fields of classical, energy efficient, secure, hierarchical, geographic, intelligent, Quality of Service (QoS)-based and multipath WSNs. However, to the best of our knowledge, no study is presented so far which may clearly categorize the routing-related survey literature for WSNs.To fill this gap, an effort is made in this paper for presenting an *in-depth* review of already published routing-related survey literature in WSNs. Our review initially proposes a generalized survey design model and afterwards analyzes the routing-related survey literature in the light of the devised *General Survey Design Framework (GSDF)*. Such an analysis describes the design soundness of the published routing-related surveys. Therefore, our review puts forth an original classification based on the *frequency-of-survey-publication* and taxonomizes the corresponding routing-related fields into high, medium and low focused areas of survey publication in WSNs. Furthermore, the surveys belonging to each main category are sub-categorized into various sub-classes and briefly discussed according to their design characteristics. On the one hand, this review is useful for beginners who may easily explore the already published routing-related survey literature in WSNs in a single document and investigate it by spending less effort. On the other hand, it is useful for expert researchers who may explore the trends and frequency of writing surveys in different areas of routing in WSNs. The experts may explore those areas of routing which are either neglected or least focused or lack in design soundness as per general survey design framework. In the end, insights and future research directions are outlined and a reasonable conclusion is put forth which may outline guiding principles for routing-related survey research in future.

## 1. Introduction

Wireless sensor networks (WSNs) are small inexpensive devices with finite energy, limited sensing/processing capability, small memory and transmission capacity [1,2]. They may perform self-organization in ad hoc mode [1,2,3] and have the ability to work autonomously. It makes them front line ally with human beings for executing various critical tasks such as elderly people health-care [4,5], structural examination [6,7], habitat monitoring [8] and near-shore environmental inspection [9]. WSNs have an indispensable role in metropolitan planning and development such as traffic handling [10,11,12,13], tunnel monitoring [14], parking lot management [15,16], water pipeline monitoring [17], water consumption examining [18], solid waste management [19], gas pipeline monitoring [20] and power grids applications [21]. In addition to that, they have a key role in security and help in intrusion detection [22], surveillance [23] and reconnaissance activities [24]. Besides that, their role in catastrophes administration is admirable such as tsunami detection and countermeasure [25], building fire monitoring [26] and disaster management [27]. The acronyms used in this survey are defined in Table 1.

Due to unique nature of WSNs, the related techniques devised for the other wireless networks were discovered unsuitable for WSNs. Eventually, new methodologies and approaches were researched and developed for WSNs keeping in view their underlying constraints, so that they may perform the assigned duties in an appropriate manner. At the inception of WSNs related research, the primary focus was energy efficiency [1,28] while throughput, delay and fairness were given secondary importance [1,28,29]. Such sensor networks were mainly employed for environmental monitoring in terms of temperature, pressure and humidity, etc. As technology flourished, the capabilities of sensor networks were further enhanced and they became able to support high data rate applications e.g., structural health monitoring requires sensor nodes to send more than 500 samples/s for recognizing any structural distortion [30]. It has also motivated the researchers to devise Wireless Multimedia Sensor Networks (WMSNs) oriented applications that may sense the delay and loss-sensitive multimedia data and relay it with high data rate to sink node using one or more routing path(s). Furthermore thanks to Wireless Power Transfer (WPT) technology that has handled the longstanding energy depletion issue in WSNs and gave birth to Wireless Rechargeable Sensor Networks (WRSNs). Such WRSNs has the ability to harvest energy and to operate in a long-lasting manner [31,32,33,34].

Apart from the different protocols operating at various layers of communication protocol stack, a variety of WSNs based routing protocols are devised that operate at network layer and focus on performance improvement and fairness in terms of energy, throughput, delay, reliability, security and so on. When a reasonable number of WSNs based routing protocols are published and relevant issues are highlighted, then the experienced researchers in this area have taken the initiative for writing a variety of worthy routing-related reviews which focus either entirely or partially on different aspects of routing in WSNs. These reviews include classical [35,36], multipath [37], energy-efficient [38,39], secure [40], intelligent [41], hierarchical [42], mobility based [43,44] surveys and so on as outlined in Table 2 and Table 3. In these surveys, the researchers have either completely or partially summarized and categorized the relevant routing protocols for WSNs and also identified the open research issues/challenges. It has helped the research community to concisely understand the particular and somehow unrevealed aspects of various areas of routing in WSNs and motivated them to further explore the unresolved routing-related challenges in WSNs in future.

Although a large number of routing-related surveys are published so far for WSNs, however to the best of our knowledge, we have not discovered any review that summarizes the routing-related survey literature in WSNs. To bridge this gap, an effort is made in this paper for analyzing and categorizing the published routing-related survey literature in WSNs. Therefore, the main contributions of this research can be delineated as follows:

- Outlining a novel *general survey design framework* that may analyze routing-related survey literature in WSNs under a variety of concrete design requirements as discussed in Section 3.
- Analyzing the published routing-related survey literature in the light of *general survey design framework* as manifested in Table 2 and Table 3.
- Presenting a novel taxonomy of routing-related surveys in WSNs which, on the basis of *frequency of survey publication*, categorizes the routing surveys literature into highly-focused, medium-focused and least-focused areas of survey publication in WSNs as shown in the organization chart in Figure 2. Furthermore the surveys belonging to each main category are sub-categorized as per the corresponding fields of publication.
- Deducing future insights and research directions as a mean of providing guidelines to beginner and expert researchers who are interested in routing-related survey research in future.

**Figure 1:**
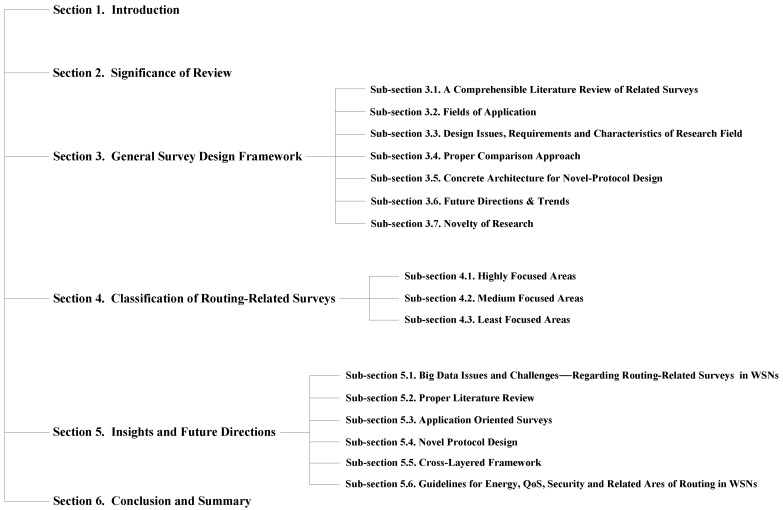
Organization of Paper.

As depicted in Figure 1, the rest of this paper is outlined as follows. In Section 2, the significance of survey is presented. The Section 3 discusses the novel general survey design framework under the perspective of survey design requirements. In Section 4, an original classification of already published routing surveys for WSNs is presented. The Section 5 put forths insights and future research directions. In Section 6, a comprehensible conclusion and summary is drawn on the basis of proposed research work.

## 2. Significance of Review

A number of routing-related surveys are published so far for WSNs which focus either completely or partially on different areas of routing in WSNs. However, the novelty and objective of this survey is to present a *comprehensive review* of routing-related survey literature for WSNs. The significance of this exhaustive research may be outlined under the following points i.e.,

- As a novel paradigm, this research manuscript provides an *in-depth* literature review of routing-related surveys in WSNs.
- The review may explore the trends of routing-related survey research in WSNs. Eventually, the researchers may figure out those areas of routing in WSNs which require more attention for survey publication in future.
- The manuscript may motivate the research community in writing surveys under the proposed general survey design framework. It may bring about coherence in routing-related survey research in future and encourages novel protocol design (please refer to Section 3.5).
- The research may supervise in identifying those areas containing the surveys of less design soundness (i.e., less count number index (CNI)) under general survey design framework and thereby require more brainstorming and attention.

## 3. General Survey Design Framework—A Perspective under Survey Design Requirements

Writing a good survey paper is quite a challenging task. It requires both in-depth knowledge and critical analysis for classifying the related research into comprehensible categories. Subsequently, meaningful conclusions can be drawn for future research and development. A good survey paper should fulfill some basic design requirements. These design requirements may provide a platform for comparing relevant reviews and organizing the survey research into intelligible categories. However to the best of our knowledge, we have not found any review that is clearly outlining such a general survey design framework for analyzing the corresponding surveys in WSNs. To bridge this gap, a general survey design framework is devised in this paper that may analytically evaluate the soundness of routing related surveys in WSNs. Subsequently, it may also serve as a *Big Data Analysis Tool (BDAT)*. The key design requirements of general survey design framework are outlined below:

### 3.1. A Comprehensible Literature Review of Related Surveys

Literature review has the pivotal role in every survey. It may inform about the ongoing and published literature and helps to bring novelty in research. However, surveying literature properly is a time consuming activity and a large number of routing-related surveys lack in proper literature review. After realizing this fact, we have decided to present a critical review of surveys having complete/partial emphasis on routing in WSNs. It may provide an *in-depth resource* of literature for the researchers who are interested in survey publication in different areas of routing in WSNs. Although *Comprehensible Literature Review (CLR)* is part of our general survey design framework, however it is deliberately not used as evaluation parameter of routing-related survey design in Table 2 and Table 3. The prime reason is that the pioneer routing-related surveys were instead the starting efforts for building the routing surveys related literature and it would be unjustified to mark them deficient in terms of the lack of proper survey related literature review. The other reason is that majority of succeeding routing-related surveys have followed the similar line and exhibited a lack of interest in properly including a routing-related surveys literature in their design. Since a comprehensive source of literature would be available after the assumed publication of this work, therefore we expect that the future routing-related surveys would consider this design metric as an integral part of their design. Subsequently, it may also help to properly develop the routing-related survey literature in different areas of routing in WSNs in future.

### 3.2. Fields of Application

By clearly outlining the relevant applications, data-delivery models and fields of implementation, a survey may capture the curiosity of both beginners and expert readers. Eventually, it may increase readability of a survey and may develop interest among readers for further research and development. The motivated readers may do more brainstorming for dealing with advanced real-world problems and for bringing forth technological innovations and advancements. Therefore, a good routing survey should properly address and explain the real-world applications of a field of research. Due to the role of *Fields of Application (FoA)* design parameter in mushrooming technology, it should be considered in routing survey design framework.

### 3.3. Design Issues, Requirements and Characteristics of Research Field

The design issues of a research field describe the challenges that a researcher may face while contributing research and development in particular field of research. On the one hand, they may cautious the researchers to be meticulous in addressing those challenges. On the other hand, they may motivate the researchers to propose novel techniques for addressing the undone issues and may contribute for the betterment of the world. In short, clearly outlining the design issue and challenges of a particular field of routing in WSNs may not only improve understanding of researchers about that field, but also stimulate novel contributions too, therefore including *Design issues, requirements and characteristics of Research Field (DRF)* in routing survey design model has a very critical role in the design completeness of a survey.

### 3.4. Proper Comparison Approach

On the basis of proper analytical and experimental comparison, the routing protocols may be differentiated into good, better and the best categories. The best algorithms may be used as a benchmark for future comparison with the newly devised techniques. Following this guideline, a routing survey may also provide a reliable and scientific mechanism for analytically and experimentally evaluating the relevant routing protocols. In this respect, a variety of parameters are used by various routing surveys for comparing the corresponding routing protocols. These parameters include energy efficiency, delay, reliability, throughput, jitter, mobility, scalability, architecture, data aggregation, security, multi-path approach and so on. However, there are some inherent challenges in analytically and experimentally categorizing the concerning surveyed protocols such as simulation set-up, operational framework and non-comparison of protocols with widely-acceptable techniques [41]. The analytical and experimental comparisons may enlighten the researchers to devise new protocols for handling the unattended issues, therefore *Proper Comparison Approach (PCA)* should be given due place in survey design framework.

### 3.5. Concrete Architecture for Novel-Protocol Design

As outlined earlier in Section 3.4 that it is very hard to compare the available protocols due to variability in their simulation set-up, operational framework and comparison approaches. As a solution to this issue, the routing-related survey should model clear-cut and acceptable framework(s) for devising, simulating and comparing new routing protocol(s) in a particular area of routing in WSNs. Such model may bring organization in in designing new routing protocols and may serve as a platform of easy comparison and evaluation in future. Since *Concrete Architecture for Novel-protocol design (CAN)* may address the daunting issue of variability in routing protocols regarding simulation set-up, operational framework and comparison with non *state-of-the-art* approaches, therefore it should be given prime importance in general survey design framework.

### 3.6. Future Directions and Trends

Outlining *Future Directions and Trends (FDT)* may provide on-hand knowledge of hot areas of research and open issues that may either need further investigation or should be considered for future research and development. A strong focus on this segment of routing survey may result into flourishing novel routing techniques that may address the pending research challenges. Since this fragment of routing survey design serves as a driving force for future research and development, therefore it should be included in general survey design framework.

### 3.7. Novelty of Research

Instead of just rearranging the surveyed routing protocols, a novel routing-related survey should provide a new approach and methodology of outlining and categorizing the surveyed literature. It describes the *pros-and-cons* of discussed routing protocols that may unearth new issues and challenges. Therefore, categorizing the routing protocols in the perspective of a new taxonomy plays an important role in increasing the knowledge resource and bringing forth new ideas and challenges for future research and development. In short, this parameter is the measure of innovativeness, uniqueness and modernity of a survey. That is why, it is also included in the survey design framework. Additionally it is worthy to mention here that the blank cells under the column *Novelty of Research (NoR)* in Table 2 and Table 3 represent those routing surveys which, as per our understanding, are less focused in contributing any substantial innovation.

## 4. Classification of Routing-Related Surveys

**Figure 2:**
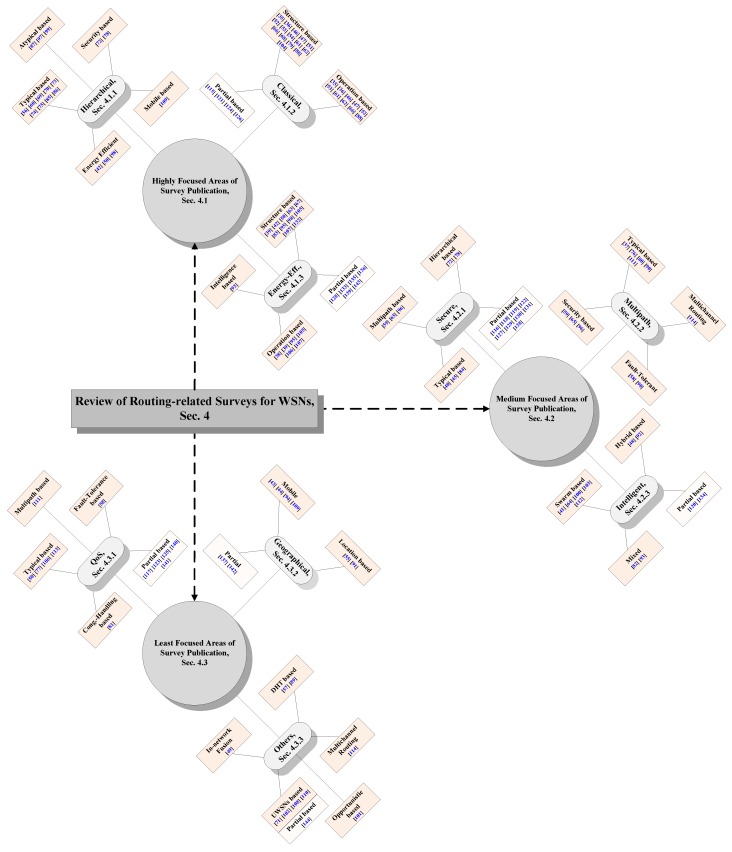
Classification of Routing based Surveys for WSNs.

The primary classification in this section is based upon 81 surveys having complete emphasis on routing in WSNs, as shown in Table 2. Since these surveys belong to different areas of routing in WSNs, therefore their *frequency of publication* is helpful in categorizing the corresponding research areas into *highly focused*, *medium focused* and *least focused areas* of survey publication as shown in Figure 2. Therefore, *field-wise publication* is the primary *evaluation metric*. Afterwards, the routing surveys belonging to each main field are sub-categorized according to their structure and mode of operation into various categories, as depicted in Figure 2. During each area-wise sub-categorization, we have also mentioned the routing surveys with partial emphasis on routing in WSNs, as outlined in Table 3. The reason for considering the partial-routing related surveys is to inform readers about them too, so that the interested readers may consult them with easiness, if required so. At the end of each sub-section, the design analysis of corresponding routing/partial-routing surveys is summarized and tabulated as shown in Table 4, Table 5, Table 6, Table 7, Table 8, Table 9, Table 10, Table 11 and Table 12. Since our focus is generalized and based on overall routing-related research in WSNs, therefore we are deliberately not going into extensive discussions about any particular area of routing in WSNs. For any specific routing field related discussions, the readers may consult the corresponding surveys as mentioned in Table 2 and Table 3. The discussions regarding various fields of classification are presented in the following sections.

### 4.1. Highly Focused Areas

The highly focused areas of routing-related surveys publication for WSNs are those having *field-wise published survey* of 14 or more as shown in Figure 2. They comprise of 18 hierarchical, 15 classical and 14 energy-efficient routing surveys that have complete emphasis routing in WSNs. Additionally, 4 classical and 6 energy-efficient surveys that discuss routing partially are also mentioned separately in Table 5 and Table 6 respectively and also in Figure 2. However, due to their partial emphasis on routing, those surveys are not counted in maintaining *field-wise publication* list. In the following text, we will briefly evaluate all surveys belonging to this category.

#### 4.1.1. Hierarchical Routing Surveys

The hierarchical routing surveys focus on cluster based routing techniques which may exhibit high efficiency for large sized networks and are scalable in nature. They are considered energy efficient and may accommodate heavy traffic load, cover large sensing area and have the ability to perform data fusion/aggregation. These surveys may be mainly classified into succeeding four categories i.e.,

*i*  In typical based categorization, the hierarchical protocols are classified on the basis of clustering procedure (such as Cluster Head (CH) election techniques, even-distribution, convergence time, clustering methodology and objectives), clustering properties (such as cluster count, size, balance, hop-count, stability, re-clustering support and inter/intra cluster topology and routing) and CH capabilities (such as type, role and mobility support).
*ii*  In atypical based categorization, the hierarchical routing protocols belonging to block, grid, chain and tree based topologies may be discussed.
*iii*  In security based categorization, security aspects of cluster-based protocols are evaluated on the basis of requirement of security goals, selection of security mechanism and prevention of security threats.
*iv*  In energy-efficient based categorization, those routing surveys are discussed which focus on energy efficiency in cluster based routing protocols.
*v*  In mobile hierarchical based categorization, those hierarchical routing protocols are discussed where either sensor or sink node or both are mobile. Such routing protocols may exhibit single-sink or multi-sink approach.

The authors in [56,60,69,70,73,74,75,85,86] have mainly classified hierarchical routing protocols into typical based categorization. In [87,97,99], the authors have classified hierarchical routing protocols using atypical based categorization. The authors in [72,78], and [42,50,98] have mainly classified hierarchical routing protocols that put forth security, and energy-efficiency based categorization respectively. The mobile hierarchical oriented categorization is exhibited by [109]. In [75], the authors have outlined a more detailed classification in terms of CH selection, cluster formation, data aggregation and communication. Additionally, the issues relevant to various phases of taxonomy are also figured out. In [99], the authors have discussed and evaluated various atypical hierarchical routing approaches in WSNs. Additionally, the application scenario of discussed protocol are outlined too. The authors in [78] devise a metric that may determine fitness of different secure hierarchical protocols to suitable applications. In [74], the authors have discussed objectives of clustering while [73] delineates routing issues and discusses both clustered and non-clustered homogeneous/heterogeneous routing protocols. In [109], the authors have discussed and compared the characteristics of various mobility-oriented hierarchical routing protocols in WSNs. The review also highlights the applications of discussed protocols that may provide guidelines for protocol development in the corresponding fields in future. The authors in [69,98] discuss the design challenges of hierarchical-based and general-based routing protocols. In [85], the efficiency of various hierarchical routing protocols is compared on the basis of numerous performance oriented parameters using Network Simulator 2 (NS-2) [145]. Cluster-oriented issue are delineated in [60] whereas general, and security-based issues are outlined in [97], and [72,78] respectively. Future research directions are discussed in [42,56,60,69,70,74,75,86,87,97,99,109]. Besides that Table 4 clearly exhibits that this taxonomy comprises of only 3 high count, 10 medium count and 5 low count surveys. Additionally, all the surveys under consideration in this category have complete emphasis on routing.

#### 4.1.2. Classical Routing Surveys

The classical routing surveys consider those routing protocols which are general in nature and do not concentrate on any specific routing domain of WSNs. Such surveys generally perform three types of routing categorizations in WSNs as described below:

*i*  In network structure based categorization, the classification metric is data centric, flat, hierarchical or location-aware.
*ii*  In network operation based categorization, the classification metric is network-flow-based, communication-model-based, QoS-aware and multipath-based.
*iii*  In partial based categorization, such classical surveys are included which although are not entirely focused on routing, however they discuss routing in a limited extent e.g., they may discuss WSNs protocols at each layer of communication protocol stack including network layer.

As far as the surveys with complete emphasis on routing are concerned, the authors in [35,36,46,47,52,53,61,62,66,88] have mainly classified routing protocols into both structural and operational based protocols while [51,54,68,79] have classified them mainly using structural based categorization. The authors in [46,52,53,54,61,62,68,79,88] have outlined design issues that should be considered while devising new protocols for WSNs. In [46,52,54], the authors have discussed various application areas of WSNs. Performance trade-offs and security challenges of data-aggregation routing protocols are outlined in [47]. The design challenges and future research directions are discussed in [35,36,46,47,51,62,66,68]. Additionally, it is evident from the Table 5 that only 1 routing survey is of high count, 9 routing surveys are of medium count and remaining 5 surveys are of low count.

The surveys having partial emphasis on routing include [115,126], and [121,124] which discuss WSNs, and WMSNs related protocols respectively. The surveys in [115,121,124] discuss various techniques at each layer of communication protocol stack including the network layer whereas [126] elaborates network and cross layer routing protocols in WSNs. The authors in [115,126], and [121,124] have outlined design issues that should be considered while devising new protocols for WSNs, and WMSNs respectively. In [115,121,124,126], the authors have discussed various application areas of WSNs, and WMSNs respectively. The design challenges and future research directions are discussed in [115,121,124,126].

#### 4.1.3. Energy-Efficiency Based Routing Surveys

Energy-efficient routing surveys consider those routing protocols which try to conserve energy for enhancing network lifetime. On the basis of reviewed literature, energy-efficient based routing surveys may be classified into following four categories i.e.,

*i*  In intelligence based categorization, energy-aware swarm intelligence based routing protocols are considered.
*ii*  In structure based categorization, energy-efficient data-centric, flat, hierarchical and location-aware routing protocols are examined.
*iii*  The operation based categorization discusses energy-efficient data-delivery-model-based, QoS-based and multipath-based protocols.
*iv*  The partial energy-efficient categorization includes those surveys whose cornerstone is not routing, however they still discuss some aspects/protocols of energy-efficient routing in WSNs such as location/connection-driven based and data acquisition based routing and so on.

As far as the surveys with complete emphasis on routing are concerned, the authors in [93] have discussed energy-aware swarm intelligence based approaches particularly Ant Colony Optimization (ACO) techniques and classified energy-sensitive routing techniques at different layers of communication protocol stack. In [39,95,105,107], the authors have discussed both structural and operational based classification of energy-aware routing protocols in WSNs. While [98] discusses energy-efficient hierarchical structure based routing protocols in WSNs. The authors in [42,50,63,67,83,132], and [38,106] have mainly discussed energy efficiency in structural, and operational based routing protocols respectively. The authors in [38] have discussed design challenges of multimedia routing techniques and limitations of non-multimedia based routing protocols. Whereas in [106], the authors have discussed those green routing protocols that focus on achieving a balance of energy efficiency and QoS in WMSNs. In [105], energy-efficient homogeneous and heterogeneous routing protocols under the static and mobile categorization are discussed. Additionally, the application of each discussed routing protocol is highlighted that may provide guidance regarding designing a routing protocol for a particular scenario. The authors in [107] have discussed various energy harvesting methods in WSNs while in [83], the authors have put forth main causes of energy wastage in WSNs. The design issues and objectives are put forth in [39,93,98], and [67,95] respectively. The authors in [38,42,83,93,105,106,107] have outlined future research challenges. Moreover, Table 6 shows that this category includes only 2 high count surveys, 8 medium count surveys and 4 low count surveys.

The surveys having partial emphasis on routing include [128,133,135,136,139,143]. Among them, ref. [128,135] discuss location-driven and connectivity-driven routing protocols in more or less the similar manner. While both [136,139] discuss some energy-efficient routing protocols in WSNs. In [143], the authors have discussed various energy-efficient structure and operation based routing protocols in WSNs. The application requirements and WSNs standards are outlined in [139] while design issues are put forth in [128,133,143]. The authors in [133] have outlined some design metrics for devising protocols in heterogeneous and fault-tolerant WSNs. However these rules are not enough for proposing a clear-cut and methodological framework like [41] that is required for devising new *state-of-the-art* protocols in the concerned area of WSNs. The authors in [128,139,143] have outlined future research challenges.

### 4.2. Medium Focused Areas

The medium focused areas of routing-related surveys publication for WSNs are those having *field-wise survey publication frequency* between 8 and 13 as shown in Figure 2. They consist of 8 secure, 10 multipath and 9 intelligence based routing survey that have complete emphasis on routing in WSNs. Moreover, 9 secure and 2 computational-intelligent based surveys that discuss routing partially are mentioned separately in Table 7 and Table 9 respectively and also in Figure 2. However, due to their partial emphasis on routing, those surveys are not considered/counted in maintaining *field-wise publication* list. Below we will briefly analyse all surveys belonging to this category.

#### 4.2.1. Security Based Routing Surveys

The security based routing surveys discuss those protocols which may carry out threat prevention, intrusion detection or hybrid approach for dealing with various security threats (such as selective forwarding, hello-flood attacks, wormhole attacks, sinkhole attacks, sybil attacks, acknowledgment spoofing, neglect and greed attacks (Denial of Service (DoS)-based)) and accomplishing various basic security objectives (such as confidentiality, integrity, availability, authentication [45] and freshness of data). According to the nature of underlying security surveys for WSNs, they may be classified into four categories as discussed below:

*i*  In multipath based categorization, the classification metric is based on threat prevention, intrusion detection, hybrid or cryptographic, key management, authentication scheme and basic security requirements.
*ii*  In hierarchical based categorization, cluster-based protocols are classified on the basis of security goals, selection of security mechanism and prevention of security threats oriented requirements and objectives.
*iii*  In typical based categorization, the secure routing protocols are differentiated on the basis of key design issues such as node deployment, energy consumption, data-reporting model, fault-tolerance, scalability, robustness, load-management, data aggregation and QoS.
*iv*  In partial secure categorization, those surveys are delineated which, apart from mainly focusing on security, also discuss routing in WSNs. Such a security oriented routing is termed as secure routing.

Considering the surveys with complete emphasis on routing, the authors in [59,65,96], and [72,78] have mainly classified routing protocols into multipath, and hierarchical based secure categorization respectively. In [40,45,84], the authors have classified routing protocols into security based typical categorization. The authors in [78] devise a metric that may determine fitness of different protocols to suitable WSNs applications. The authors in [45,59] have put forth threat model while [40] employs key design issues of routing in WSNs that may help to compare secure multipath routing protocols. The threat model proposed in [59] is helpful in identifying the intentions and schemes of antagonist and the security issues in routing process. Such identifications may help the protocol designers to address them in a proper manner. However the survey does not provide any clear-cut and methodological framework like [41] that is helpful in *state-of-the-art* secure multipath routing protocols in WSNs. The surveys in [40,65,72,78,84,96] have discussed security issues and requirements of WSNs. Future research directions are outlined in [40,59,84]. Furthermore, Table 7 figures out that this classification includes only 2 high count, 5 medium count and 1 low count surveys.

The surveys having partial emphasis on secure routing consist of [116,118,119,122,127,129,130,131,138]. The authors in [116,118,119,122,127,129,130,131,138] have discussed some aspects of secure routing and therefore, such surveys are classified into security based partial categorization. The authors in [129,130,131,138] have discussed attacks and counter measures at different layers of communication protocol stack including network layer. The surveys in [118,119,122,127,130,131] have discussed security issues and requirements of WSNs. Future research directions are outlined in [116,118,119,127,130,131].

#### 4.2.2. Multipath Based Routing Surveys

The multipath based routing surveys consider those routing protocols which employ multipath approach for sending data between source and destination and may provide load balancing, reliability, error resilience, interference avoidance and security. Such surveys can be classified into three groups as discussed below:

*i*  In typical multipath categorization, the routing metric may be path disjointedness based, path selection based, path reliability oriented, path maintenance based or infrastructure/non-infrastructure based and so on.
*ii*  In multichannel routing based categorization, the classification metric is JOINT and DISJOINT channel assignment and routing in multichannel WSNs.
*iii*  In security based categorization, the classification metric is threat prevention, intrusion detection or hybrid based.
*iv*  In fault-tolerant based categorization, the classification metric is retransmission and replication based.

The authors in [37,76,80,90,111] have mainly classified routing protocols using typical multipath categorization. In [114], the authors have discussed both multipath and single path multichannel routing in WSNs. The authors in [59,65,96] have classified multipath protocols using security based classification while fault-tolerant based categorization is employed in [58]. In [111], the authors have discussed and compared the multipath routing approaches with QoS guarantee in real-time WMSNs. The authors in [114] have discussed multichannel-related issues in WSNs. The authors in [59] have outlined a threat model for performing secure multipath routing while in [65], the authors have discussed multipath protocols by devising a security matrix. The security issues are outlined in [59,65] whereas routing issues and challenges are discussed in [37,80,111]. In [96], the authors discuss security issues/requirements and attacks in WSNs. Future research directions are outlined in [58,59,76,111,114]. The characteristic and elements of multipath routing are put forth in [90]. The authors in [76] have theoretically explained some basic rules for designing multipath routing protocols in WSNs. Nevertheless it is lacking in a clear-cut and methodological framework like [41] which may be instrumental in formulating new *state-of-the-art* multichannel routing protocols in WSNs. Furthermore, it is noticeable from Table 8 that this organization accommodates 4 high count, 4 medium count and 2 low count surveys.

#### 4.2.3. Computationally-Intelligent Based Routing Surveys

The Computationally Intelligent (CI) based surveys analyze those routing techniques which have the ability to adapt themselves with dynamic and challenging environments. These surveys may be classified into four categories as outlined below:

*i*  In swarm intelligence based categorization, the robustness, reliability and flexibility of optimization techniques such as shortest-path based Ant Colony Optimization (ACO), foraging based Bee Colony Optimization (BCO), Particle Swarm Optimization (PSO), Termite Colony Optimization (TCO) or Spider Monkey Optimization (SMO) are discussed.
*ii*  In hybrid intelligence based categorization, the adaptability, steadfastness and strength of both already discussed swarm intelligence and adaptive intelligence based techniques (such as knowledge-base/experience based Reinforcement Learning (RL), human reasoning oriented Fuzzy logic (FL), genetic-selection/natural exclusion based Genetic Algorithm (GA), human neurons based Artificial Neural Networks (ANNs), morphogenesis-based Reaction Diffusion (RD) and bacterial-signaling-based quorum sensing (QS)) are evaluated.
*iii*  In mixed based categorization, both biologically-inspired or non-biological routing techniques may be discussed and compared.
*iv*  In partial based categorization, those surveys are delineated which consider CI based routing as a subpart of their discussion regarding intelligence based WSNs.

As far as the surveys with complete emphasis on routing are concerned, the authors in [41,64,100,103,112] have mainly discussed routing protocols belonging to swarm intelligence based categorization. In [48,92], the authors have mainly discussed various biologically inspired hybrid intelligence based routing techniques. The authors in [82,93] have mainly discussed routing protocols belonging to mixed based categorization. In [103], the authors have discussed the latest swarm-intelligence based routing approaches. The paper very briefly outlines some aspects of TCO whereas the functionality of SMO is also summarized in a concise manner. In [48], the authors have discussed mathematical principles of biological calculations and general routing framework regarding WSNs. The authors in [41,100] delineates various design challenges for routing in WSNs. Additionally, reference [41] outlines a general framework that may provide guidelines to design new swarm intelligence based routing protocols and also delineates a useful discussion that describes shortcomings in the already published research work. The design issues and challenges are discussed in [48,64,82,103]. Future research directions are summarize in [41,48,82,103,112]. Furthermore, Table 9 manifests the presence of 3 high count surveys, 5 medium count surveys and only 1 low count survey.

The surveys having partial emphasis on routing consist of [120,134]. In [120], the authors have mainly discussed various ant colony optimization based routing approaches in WSNs. The survey in [134] is more comprehensive and evaluates computational intelligence based energy aware routing and clustering in WSNs. The design issues and challenges are discussed in [134]. Future research directions are summarize in [134].

### 4.3. Least Focused Areas

The least focused areas of routing-related surveys publication for WSNs are those having *field-wise survey publication of 7 or less* as shown in Figure 2. They may include 7 QoS, 6 Geographic and 9 other surveys (that include in-network fusion oriented routing, distributed hash table (DHT) based routing, Underwater Wireless Sensor Networks (UWSNs) based routing, opportunistic routing and multichannel routing) that have complete emphasis on routing in WSNs. Additionally, 2 geographic, 5 QoS and 1 UWSNs oriented surveys that discuss routing partially are also mentioned separately in Table 10, Table 11 and Table 12 and also in Figure 2. However, due to their partial emphasis on routing, such surveys are not counted towards maintaining *field-wise publication* list. The surveys belonging to this category are briefly examined below.

#### 4.3.1. QoS Based Routing Surveys

The QoS-based routing surveys consider those routing protocols which struggle to maintain a balance between energy consumption, data quality [81], and quantity. That is why, QoS-based surveys focus on energy efficiency, reliability, end-to-end delay minimization, fault tolerance, throughput/bandwidth enhancement, delay-variability diminution, and collision/congestion avoidance [81]. The available QoS-based routing surveys may be broadly classified into subsequent categories i.e.,

*i*  In typical QoS-based categorization, routing surveys may reckon those QoS protocols that consider a broad-spectrum of QoS routing in WSNs. These QoS protocols may consider the parameters such as energy efficiency, reliability, packet-delivery rate, end-to-end delay, network lifetime, throughput, protocol overhead and robustness.
*ii*  In multipath-QoS based categorization, those multipath routing protocols are discussed which provide QoS assurance such as reliability to WSNs.
*iii*  In fault-tolerance based categorization, those routing protocols are discussed which have the ability to work continuously even in the presence of faults for increasing reliability and availability.
*iv*  In Congestion (Cong.)-handling based categorization, those routing protocols are delineated which have the capability to detect, control and avoid congestion by handling issues such as channel contention/overload, packet collision, buffer overflow, data-rate control and load balancing near sink node.
*v*  In partial QoS-based categorization, those surveys are delineated which consider typical, fault-tolerant and congestion-oriented QoS-based routing as a subpart of their discussion regarding QoS-based routing protocols in WSNs.

While considering the surveys with complete emphasis on routing, the authors in [50,77,106,113] have mainly classified routing protocols employing typical QoS-based categorization. In [106], the authors have discussed those WMSNs protocols that try to maintain a balance between QoS and energy efficiency. In addition to discussing QoS-aware routing approaches, the authors in [113] have outlined the distributions of QoS-oriented literature and QoS parameters used in the literature. In [111], the authors have discussed and compared the multipath routing approaches guaranteeing QoS in real-time WMSNs. The authors in [58] have categorized routing protocols using fault-tolerance based categorization while [81] have mainly classified routing protocols applying QoS-based congestion-oriented categorization. In [77], a QoS-based comparison of discussed routing protocols is presented using NS-2 [145]. The authors in [77,81] have outlined challenges of QoS-based routing in WSNs. In [113], QoS issues in WSNs are elaborated while QoS modeling requirements of WMSNs are delineated in [111]. Future research directions are outlined in [58,81,106,111,113]. Besides that Table 10 clearly demonstrates the presence of 2 high count, 4 medium count and 1 low count surveys.

The surveys in this category with partial emphasis on routing include [117,123,125,140,141]. The article [125] also outlines a case study for forest fire surveillance and thereby, is helpful to enhance practical learning. The authors in [117,123,140] have discussed the issues of QoS-based routing in WSNs. Moreover, future research directions are presented in [117,123,140,141].

#### 4.3.2. Geographic Based Routing Surveys

These surveys consider such routing protocols where sensor nodes have either Global Positioning System (GPS) or some location-aware system that may help to provide awareness about their position. On the basis reviewed literature, these protocols may be classified into three categories as discussed below:

*i*  In mobile based categorization, such surveys are discussed which consider mobility as main focus and categorize routing protocols on the basis of network structure (such as grid-based, cluster-based, tree-based, zone-based), state of information (such as proactive-based, reactive-based, hybrid-based), energy-efficiency (such as power control and saving, load-distribution-based), mobility (sink-only, node-only, hybrid) and biologically inspired routing.
*ii*  In location-based categorization, the classification metric may be flooding-based, curve-based, grid-based, geography-based and trajectory-based while mobility is not generally focused.
*iii*  The partial geographical categorization includes those surveys which consider some aspects/protocols of geographic routing in WSNs.

In case of the surveys with complete emphasis on routing such as [43,44,94,109], the routing protocols are categorized by employing mobility based classification. The protocols in [43,44] consider only mobile sink based routing protocols whereas [94,109] considers mobility in general. The authors in [55] have mainly classified routing protocols using location-based categorization while [91] delineates brief discussion regarding location-based routing protocols in WSNs. In [109], the authors have discussed and compared the characteristics of various mobility-oriented hierarchical routing protocols. Highlighting the applications of discussed protocols is helpful in protocol development in the corresponding fields in future. In [43,91], the authors have discussed challenges of location-based and mobility based routing respectively. The authors in [94] have discussed general issues of mobility in WSNs. Future research challenges are delineated in [43,44,55,94,109]. Moreover, it is apparent from Table 11 that this stratification contains 3 high count, 2 medium count and 1 low count surveys.

The surveys having partial emphasis on routing include [137,142]. In [137,142], the authors have discussed routing protocols under restricted geographical categorization. The authors in [142] have described some micro-mobility oriented routing protocols which may or may not consider triangular routing in their operations. The authors in [137] have discussed general issues of mobility in WSNs while [142] outlines design issues and challenges of mobility management in 6LoWPAN based WSNs. Future research challenges are delineated in [137,142].

#### 4.3.3. Other Routing Surveys

The authors in [49] have discussed in-network fusion techniques under routing-driven, coding-driven, and fusion-driven categories. The authors in [57,89] have discussed distributed hash table (DHT) based routing in WSNs. In [57], a general model of DHTs-based routing is presented while [89] delineates a more comprehensive classification of DHT based routing protocols in WSNs which consider structure, identifier-assignment and auto-deployment of concerned protocols. Moreover, it is clear from Table 12 that this categorization embodies only one high count survey and three medium count surveys which clearly exhibits a great potential of research even in these least focused areas of survey publication.

In [71], the authors have discussed the functionality of various routing protocols for underwater wireless sensor networks (UWSNs) and classified them mainly into structural and operational based categories. The author in [102] have discussed and compared various non-cross-layered traditional cross-layered and intelligent cross-layered routing protocols in UWSNs. The authors in [108] have discussed the advantages and disadvantages of various data forwarding routing approaches in UWSNs. In [110], the authors have discussed various localization-oriented and localization-free routing protocols for UWSNs. In [144], the authors have evaluated various UWSNs based MAC and Routing protocols using comparison and simulation mechanisms. The design issues and challenges are discussed in [71,102,108,110,144]. Future research directions are outlined in [71,102,108,144].

In [101], the authors have discussed and compared some opportunistic routing protocols in WSNs. Moreover, some brief future directions are also outlined. The authors in [114] have extensively discussed numerous single/multi-path and single/multi-radio multichannel routing protocols in WSNs under a novel taxonomy and summarize the pros and cons of discussed protocols. It discusses various applications of multichannel routing, design issues/challenges and future research directions in a comprehensive manner. This information is very useful for providing a thorough information regarding understanding the multichannel routing approach and designing novel multichannel routing protocols in WSNs.

## 5. Insights and Future Directions

Quite a handsome number of surveys are published so far that emphasize on routing in WSNs. These surveys categorize the relevant routing protocols based on various classification parameters and highlight the underlying technological issues, advancements and future research directions. However, to the best of our understanding, there is still a need to do more routing-related survey research for WSNs in future under the following insights and guidelines.

### 5.1. Big Data Issues and Challenges—Regarding Routing-Related Surveys in WSNs

As per IBM definition, big data should have three characteristics namely volume, velocity and variety [146]. Here, volume represents data quantity, velocity corresponds to data speed and variety quantifies richness in terms of data type. As per our cognizance, reviewing routing-related surveys in WSNs meets the above stated big data dimensions in a moderate sense. Therefore like big data, routing-related surveys in WSNs may suffer from a variety of issues that correspond to data comprehension, analysis, organization, management and so on as described below:

#### 5.1.1. Data Comprehension Issue

Data comprehension and understanding may help to properly arrange and represent data [147]. Therefore, it is a primary requirement in dealing with large volumes of data. A reasonable data arrangement may add value to data and is helpful in proper analysis and decision making. If the data is not properly arranged, then it is quite challenging to assign any proper value to it. However, it is still possible to analyze the unstructured data by using various advanced softwares such as Hadoop, etc. Prior to this work, no study is published so far that may focus on understanding and comprehending the routing-related surveys in WSNs. To bridge this gap, an effort is made in this paper to arrange, comprehend and understand the routing-related surveys in WSNs. It may help to assign value to various routing-related surveys in WSNs based on either general survey design framework as outlined in Table 2 or the frequency of survey publication regarding a particular area of routing in WSNs as depicted in Figure 2. However, still it is a pioneer step in this regard and more research is needed in future.

#### 5.1.2. Data Analysis and Processing Challenge

Due to ongoing research in various fields of technology, data volume is continuously increasing day-by-day which requires more and more space for data storage. If such a big data is properly analyzed, then it can be a source of utility and knowledge resource for future decision making. Otherwise, it is simply a storage burden which may continuously consume system resources. Therefore, there is a need to devise novel data analysis frameworks, so that the real benefits of data storage may be ascertained. Performing proper data analysis may in turn raise some thought-provoking questions such as the definition of proper/improper analysis in a given scenario, relevancy/irrelevancy of data for analysis, choice of post-analysis decision making approaches and so on. To the best of our knowledge, we have not found any data analysis model for organizing routing related surveys in WSNs.

Since each routing related survey for WSNs discusses a variety of relevant protocols, therefore analyzing and categorizing a large amount of such routing-related surveys is a tedious and time consuming problem. To address this issue, two frameworks are proposed herein. The first one is the general survey design framework as discussed in Section 3 that may help to analyze the design soundness of routing-related surveys in WSNs. The second framework may help to examine the routing-related surveys on the basis of frequency of surveys publication as considered in Section 4. Such analysis may help to clearly categorize and index the routing-related survey literature into various categories and uncover those areas which are either least-focused, neglected-altogether or unsatisfactorily-managed and require further attention for bringing about technological advancements in WSNs. For example:

- Least focused areas of routing surveys publication in WSNs may include in-network fusion [49], DHT based routing [57,89], UWSNs oriented routing [71,102,108,110] and opportunistic based routing [101].
- The neglected-altogether areas include those that are NOT in-depth reviewed at all. One such example is multichannel routing in WSNs where to the best of our knowledge, only one survey [114] is written very recently.
- The unsatisfactorily-managed areas include those where, as per proposed GSDF in Section 3, the surveys of low quality are published as illustrated in Table 2 and Table 3.

Therefore, more research and brain storming is immensely required in the above mentioned areas of routing in WSNs. It may result into devising new solutions and exploring novel dimensions for dealing with the outstanding challenges regarding different areas of routing in WSNs and bringing forth further technological developments in those fields.

#### 5.1.3. Data Organization and Management Issue

The primary focus of big data user is data quality [148] rather than data quantity. High quality oriented extensive amount of data is helpful in deducing reasonable and credible conclusions about the available data [148]. Data quality can be accessed on the basis of data accuracy and timeliness. Data qualification and verification is helpful in filtering the desired data from the data pool. Such a filtered data may be reliable in a given context and helpful in executing data investigation related activities.

Data qualification and verification has a very important role in data organization and management. Once the data is well qualified and properly analyzed, then it becomes easy to organize and manage it. To the best of our knowledge, we have not found any particular data organization and management model for handling routing related surveys in WSNs. However, this gap is abridged in this survey whereby the proposed general survey design framework is used to organize the routing related surveys in WSNs into into various categories such as high, medium and low count number index based surveys as shown in Table 2 and Table 3. Whereas, the frequency of survey publication is employed to categorized the routing-related surveys in WSNs into highly, medium and least focused areas of survey publication as depicted in Figure 2. However, there is a need to do further research for making headway regarding progress of big data analysis.

#### 5.1.4. Quality Assurance and Quality Evaluation

Properly organizing and managing the data may result into achieving data coherence and consistency. Such a coherence may assist in systematic quality assurance and quality evaluation of data. Consequently, it becomes easier in drawing logical conclusions and proposing future research directions about data. Following these guidelines, the routing-related survey literature in WSNs are organized and managed by employing general survey design framework (Section 3) and frequency of survey publication (Section 4). It may motivate the researchers to perform two-pronged activities i.e.,

*(i)*  Encourage researchers to write novel routing surveys under the aforementioned guidelines in Section 3. Such guidelines may not only provide coherence and consistency in the design of future routing-related surveys in WSNs, but also make their comparisons easy.
*(ii)*  Motivate researchers to evaluate the the quality of already published routing surveys and classify them into different categories on the basis of their design soundness. Furthermore, explore those surveys that exhibit low quality design and improve their quality by publishing novel *state-of-the-art* surveys.

#### 5.1.5. Data Progress Challenge—in terms of Data Depth and Data Breadth

For keeping abreast with the ongoing research, there is a need to evolve and develop data with time. Such data evolution and development may either be data-revision-based or data-innovation-oriented. Here, data-revision-based development corresponds to increase in data breadth only by considering the already devised classifications, techniques and methodologies from the past. Whereas data-innovation-oriented evolution is akin to enhancement in data depth and richness that may be achieved by conceiving novel taxonomies, perspectives and approaches. In addition to that, properly handling both data-revision and data-innovation based approaches is quite a tedious and time consuming issue. Following this issue to the best of our understanding, we have employed an attribute entitled as Novelty of Research (NoR) in Section 3.7 of our GSDF. Additionally on the basis of NoR, we have evaluated the published routing-related surveys in WSNs as shown in Table 2. However, there is still a need to do more research in this regard and to present such frameworks that may help to clearly differentiate between data depth and breadth.

#### 5.1.6. Data Diversification Issue

Data may be structured or unstructured in nature. The unstructured data is unsystematic and unrefined [149], therefore it is costly to deal with it [149]. That is why, relying completely on unstructured data for decision making may result into inappropriate conclusion. Conversely, the structured data is well ordered, reasonable and systematic [149] and thereby contains only the relevant information. Although such information is helpful in decision making, however it ignores the underlying hidden information in unstructured data and may produce inappropriate results. In this survey, we have clearly structured a large number of routing-related surveys in WSNs, however we leave the question of identifying and handling the unstructured data (regarding routing-related surveys in WSNs) for future research and investigation. We may anticipate that like the big data, it may be analyzed by *Big Data Analysis* software such as Hadoop, etc. for providing the most realistic outcomes. It may also be a future research direction too.

#### 5.1.7. Data Skills Acquisition Challenge

Big data science is a new technological platform that requires a variety of expertise and skills such as investigation, modeling, designing and interpretation. Such skills can be learned through a process such as formal higher education, special training workshops and research/creativity-oriented environment [149]. For immediate and proper solution, the big data scientists/architects/analysts may be hired or consulted for appropriate investigation, analysis, organization and management of big data. Such experts may easily guide in exploring the hidden patterns in unstructured data that may otherwise be very difficult to discover. However, still there is a need to devise a clear framework for big data skills acquisition, collaboration and management that may be fruitful in progressing big data analysis and eventually is an open research direction for future.

### 5.2. Proper Literature Review

Proper literature review provides up-to-date knowledge regarding a particular field of research. It may help the researchers for exploring the unaddressed issues and to brainstorm them further for devising novel solutions. In this way, proper literature review has a key role in bringing about data-depth, data-richness and data-novelty. However, as per routing-related reviewed literature for WSNs, it is quite evident that a large number of routing surveys are lacking in proper literature review. To bridge this gap, an extensive literature review of routing-related surveys is provided in this paper that may serve as a first-hand knowledge-resource in this regard. It may help to evaluate the trends of research community in publishing routing-related surveys in different areas of WSNs. Such trends may be critically evaluated in future under the available literature and may bring forth more interesting results. Critically evaluating these trends on the basis of novel numerical methods may further contribute in progressing big data analysis. Consequently on the one hand, the routing-related surveys would be properly anatomized, organized and managed. Whereas on the other hand, the big data analytics and management tools would be further developed that may be also used for analyzing the data in relevant areas of research.

### 5.3. Application Oriented Surveys

A variety of routing-related surveys are published so far for WSNs that may focus on various performance aspects such as energy efficiency, security, intelligence, mobility, in-network processing and so on. There are a variety of routing-related surveys that may discuss the fitness of discussed protocols regarding various application areas in WSNs e.g., in [46,52,54], the authors have discussed various application areas of WSNs. The authors in [78] have devised a metric that may determine fitness of different secure hierarchical protocols to suitable applications. In [99], the application scenario of atypical hierarchical routing approaches in WSNs is presented. In [109], the authors have highlighted the applications of discussed mobility-oriented hierarchical routing protocols in WSNs. In [105], the application of each energy-efficient homogeneous and heterogeneous routing protocol is highlighted. The authors in [114] have extensively discussed the applications of multichannel routing in a comprehensive manner. However to the best of our knowledge, we have not found any routing-related survey in WSNs that may in-depth and dedicatedly analyze the role of routing protocols in performing an application in WSNs. Therefore, there is still a need to publish such routing-related surveys that may highlight the role of WSNs oriented routing protocols in developing critical applications such as water conservation, irrigation, target tracking, border management and so on. On the one hand, the application-specific routing surveys may highlight the indispensable role of routing protocols in bringing forth technological advancements in WSNs. On the other hand, they may bring forth the growing role and necessity of WSNs in bringing comfort in the lives of human beings. Eventually, they may create interest among the readers/ researchers and motivate them to contribute more in this regard.

### 5.4. Novel Protocol Design

Each routing survey evaluates and analyzes a variety of routing protocols. Such an analysis is beneficial in deducing proper guidelines for devising new routing protocols as per corresponding area of routing in WSNs. The routing protocols designed on the basis of those common guidelines may show similarity in their design pattern which may make their analytical and experimental comparison simpler. Therefore, the routing-related surveys in WSNs should consider this aspect seriously. It would be more appreciated, if a general routing protocol design framework would be devised that may provide proper insights and guidelines for designing a general routing protocol in WSNs. Such a framework may help in handling the challenge of variability among routing protocols in the context of simulation setup, operational framework and comparison with *state-of-the-art* approaches.

### 5.5. Cross-Layered Framework

Contrary to strict layering approach of Open Systems Interconnection (OSI) model, the Cross-Layered (XL) approach allows interaction among various layers of communication protocol stack which may result into enhancing system performance [150,151,152]. Although the authors in [126] have reviewed network and cross layer routing protocols in WSNs while the authors in [102] have explored traditional/intelligent cross-layered routing protocols in UWSNs. However to the best of our understanding, there is a need to publish more routing-related surveys in WSNs that may focus on the synergies between network and other layers of *communication protocol stack*. Such XL routing surveys may eventually serve as a valuable resource for featuring the benefits of effective routing for solving various real world challenges in WSNs. They may also discuss and highlight the design challenges, methodologies and benefits of employing XL routing approaches in WSNs and may guide the researchers for successfully employing such XL routing approaches in their future research projects. However, XL approaches may suffer from various issues [153] and design intricacies [154] too, therefore XL oriented surveys should also discuss the inherent design complexities and relevant solutions for achieving high performance in WSNs.

### 5.6. Guidelines for Energy, QoS, Security and Related Areas of Routing in WSNs

Due to crucial role of WSNs in our lives, their popularity is increasing day-by-day. The WSNs may either be static or dynamic in nature. The static WSNs are immovable and perform sensing/monitoring activities by permanently occupying the assigned spaces. The example is structural health monitoring [6] or underground train tunnels investigation [14]. The dynamic WSNs consist of moving nodes such as surface-robots or air-drones that may perform surveillance and reconnaissance of critical installations. The example includes fire investigation [23], borders, bridges and dams monitoring. Both static and dynamic WSNs are affected by a variety issues as discussed below:

- Energy conservation is the key goal in the design of routing protocols for both static and dynamic WSNs. In case of static WSNs, the immediate neighbors to sink suffer more readily from hot-spot issues due to network traffic. On the other hand, dynamic WSNs may suffer from additional energy consumption due to mobility and frequent connection/data loss. Although mobile sink and multipath-routing approach are promising solutions for achieving energy conservation [43] in WSNs. Another promising direction may be dynamic multi-sink multipath approach for achieving energy efficiency in WSNs which requires in-depth review and analysis in future.
- A variety of energy conservation approaches are devised for WSNs. However, a sensor network with no indigenous source of energy harvesting is destined to die down due to energy depletion of sensor nodes. A solution is to employ such energy harvesting mechanisms that may provide a constant source of energy assistance to WSNs and may help in averting the danger of dying out of sensor network due to energy exhaustion. However, the prevailing energy harvesting machinery is bigger than the small-sized sensor nodes [107] and is expensive to generate usable power for sensor nodes [107]. Therefore for handling the constant nuisance of power exhaustion in sensor networks, there is a tremendous need of doing further research for devising such miniature nano-scale energy harvesting equipments that may easily generate the required energy for recharging sensor nodes and help sensor network for functioning continuously.
- Hierarchical routing helps to achieve energy efficiency in WSNs [109]. However it may suffer from various issues such as optimum cluster size, cluster head selection/communication and cluster topology/scalability, etc. These issues become more challenging in case of mobility-oriented clustering where network topology frequently changes which may seriously impact network connectivity. Eventually, clustering would be severely affected. In case of high speed node mobility, then majority of network resources would be consumed in cluster formation and upgradation rather than desired data transmission. Therefore there is a need to survey the issues and challenges of mobility-oriented clustering in WSNs.
- Both energy and QoS are closely associated e.g., increasing the packet transmission energy may increase the transmission range of a packet. Eventually, less hops are required to send data to destination which may decrease end-to-end delay [38]/jitter and improve system reliability. However, such mechanism may more readily drain the energy of sensor nodes and eventually cause early death of sensor network [38]. One solution to handle such energy drainage may be to use energy harvesting nodes for this purpose. However when more nodes may send packets with high energy simultaneously, then they may disrupt each other communication. A solution may be to use adaptive power control, but it may soon induce power competition among sensor nodes. Since energy-aware QoS routing assures required bandwidth, latency and energy-efficient routes [36], therefore it should be more thoroughly surveyed along with underlying issues and challenges for bringing efficient and more realistic solutions in this regard.
- Employing security along with routing requires additional energy, storage and processing capability. Security mechanisms may induce processing delays and bandwidth loss in WSNs which may seriously impact QoS-routing in WSNs. Therefore it is desirable to write such routing surveys that may highlight the issues, applications and comparisons of security and QoS [130] oriented routing in WSNs. Such surveys may discuss state-of-the-art secure-QoS routing approaches and highlight future research directions in this regards.
- Secure routing improves reliability of both static and dynamic WSNs. Since traditional cryptography and network security techniques are infeasible for secure routing in WSNs, therefore there is a need to analyze and survey novel featherweight multi-factor authentication and authorization approaches and security mechanism in WSNs.
- Although it is more energy efficient to-process than to-transmit the same amount of data [155], however it is also a fact that data fusion complicates secure-routing design [35], introduces delay [49] and compromises reliability [49]. Therefore, there is a need to extensive survey the issues, challenges and comparisons of data fusion and secure-QoS oriented routing in WSNs.
- Since, single-sink oriented routing techniques under-perform than multi-sink based routing approaches [84], therefore there is a need to in-depth survey those routing techniques that implement multi-sink approach in WSNs. The advantages of multi-sink oriented routing may be further highlighted under energy, security, QoS and scalability constraints for achieving high performance in WSNs.
- The computational intelligence based routing approaches such as biologically inspired (e.g., ant based, bee based, particle swarm, termite colony and spider monkey based optimization), machine learning, genetic algorithm, fuzzy logic, evolutionary computing, reinforcement learning and artificial neural networks should be reviewed in more depth. From the recent past, the researchers have started applying these techniques for routing in WSNs and they have exhibited promising results. However, there is a need to survey various aspects of these techniques more deeply for achieving further technological advancements in WSNs.
- The multichannel routing approach may allow sensor nodes to use a variety of orthogonal channels for sending data to destination. It may ensure parallel communications [156], decreases delay [156,157] and increases throughput [156,157] in WSNs. However, it requires additional resources in the form channel scanning, channel decision and channel switching, etc. which may require additional energy for providing the desired QoS in WSNs. Only one multichannel routing survey [114] is published so far for WSNs that may put forth a novel taxonomy for classifying and analyzing the single/multi-path and single/multi-radio multichannel routing protocols in WSNs into JOINT and DISJOINT categories. Although the survey brings forth the applications, prevailing issues and future research challenges of multichannel routing in WSNs in a detailed manner, however still there many dimensions in which novel multichannel routing surveys can be written e.g., one of such dimension is the mobile multi-radio multichannel routing in WSNs where to the best of our knowledge no multichannel survey is written so far. Additionally, the pros and cons of multichannel routing protocols may be critically surveyed in-terms of energy-efficiency and QoS using various analytical and simulating mechanisms for unveiling further hidden aspects of this area of research.

## 6. Conclusions and Summary

Coherence provides logical connectivity among ideas and serves as a source of organization. Coherence in the design of WSN related routing surveys may not only make their comparisons easy, but also help in deducing logical conclusions that may serve as a source of technological advancements in WSNs. For achieving such a coherence, we have undertaken a pioneer step in proposing a novel *general survey design framework*. The proposed framework is helpful in drawing methodological and concrete guidelines that, on the one hand may advice beginners in writing routing-related surveys in future. Whereas on the other hand, they may assist experts in evaluating the design soundness of published routing-related surveys in WSNs. Furthermore, it may motivate experts in proposing concrete architecture for novel protocol design in the concerned areas of routing in WSNs. Eventually, it would enforce organization in routing protocols in terms of simulation set-up, operational framework and comparison criteria that is required in handling any undue variability in the design of new routing protocols in WSNs. Due to generality of proposed novel survey design architecture, it may be applied to the related fields of technology too. Besides that, the novel *general survey design framework* may evaluate the design soundness of already published routing-related survey literature in WSNs. Such categorizations may motivate the interested researchers to do more brainstorming in those areas of routing-related survey publication which are either neglected, deficient or exhibit low design soundness. Consequently, technological maturity and development is achieved.

Based on the frequency of survey publication in various areas of routing in WSNs, this manuscript takes a novel initiative in classifying the published routing surveys in WSNs into various categories such as *highly-focused*, *medium-focused* and *least-focused* areas of survey publication. In the end, our review also provides enormous insights and future guidelines to beginner and expert investigators that are helpful in writing routing-related surveys in the unattended or least focused areas of routing in WSNs. Subsequently, both beginner and expert researchers may choose a field of routing-related survey research with less difficulty and may easily contribute with novelty to the survey oriented knowledge resource.

## Figures and Tables

**Table 1:** Listing of acronyms with description.

Acronyms	Description	Acronyms	Description
ACO	Ant Colony Optimization	ANNs	Artificial Neural Networks
BCO	Bee Colony Optimization	BDAT	Big Data Analysis Tool
CAN	Concrete Architecture for Novel-protocol design	CH	Cluster Head
CI	Computationally Intelligent	CLR	Comprehensible Literature Review
CNI	Count Number Index	DHT	Distributed Hash Table
DoS	Denial of Service	DRF	Design issues, requirements and characteristics of Research Field
FDT	Future Directions & Trends	FL	Fuzzy Logic
FoA	Fields of Application	GA	Genetic Algorithm
GPS	Global Positioning System	GSDF	General Survey Design Framework
Hg	High (value of CNI)	Lw	Low (value of CNI)
Md	Medium (value of CNI)	NS-2	Network Simulator 2
NoR	Novelty of Research	OSI	Open Systems Interconnection
PCA	Proper Comparison Approach	PSO	Particle Swarm Optimization
QoS	Quality of Service	QS	Quorum Sensing
Rk	Rank (based on CNI)	RD	Reaction Diffusion
RL	Reinforcement Learning	SMO	Spider Monkey Optimization
TCO	Termite Colony Optimization	UASNs	Underwater Acoustic Sensor Networks
UWSNs	Underwater Wireless Sensor Networks	WMSNs	Wireless Multimedia Sensor Networks
WPT	Wireless Power Technology	WRSNs	Wireless Rechargeable Sensor Networks
WSNs	Wireless Sensor Networks	XL	Cross-Layered

**Table 2:** Design analysis of surveys (with complete emphasis on routing in WSNs) under GSDF.

Surveys	FoA	DRF	PCA	CAN	FDT	NoR	CNI	Rk
Karlof et al. [45], 2003		✓				Two types of novel attacks in WSNs, Security analysis routing protocols	2	Lw
Al-Karaki et al. [35], 2004	✓	✓	✓		✓	Network-structure and protocol- operation based taxonomy	5	Hg
Akkaya et al. [36], 2005	✓	✓			✓	Network-structure and protocol- operation based taxonomy	4	Md
Yang et al. [46], 2005	✓	✓			✓		3	Md
Rajagopalan et al. [47], 2006	✓	✓			✓	Network structure and operation oriented data aggregation	4	Md
Iyengar et al. [48], 2007	✓	✓			✓		3	Md
Luo et al. [49], 2007	✓		✓		✓	Data-fusion based routing/ coding/ fusion driven protocols	4	Md
Shafiullah et al. [50], 2008			✓				1	Lw
Wan et al. [51], 2008		✓			✓		2	Lw
Boukerche et al. [52], 2008	✓	✓	✓				3	Md
Garcia et al. [53], 2009		✓	✓			Optimized routing approaches invented Spanish Universities	3	Md
Biradar et al. [54], 2009	✓	✓	✓				3	Md
Jin et al. [55], 2009					✓		1	Lw
Jiang et al. [56], 2009			✓		✓	Features of clustering algorithms in WSNs	3	Md
Thanh et al. [57], 2009		✓	✓			Distributed hash table based routing in WSNs	3	Md
Alwan et al. [58], 2009			✓		✓	Fault-tolerance based routing in WSNs	3	Md
Stavrou et al. [59], 2010	✓	✓	✓		✓	Threat model describing the aims and schemes of adversary Taxonomy of secure-multipath routing in WSNs	5	Hg
Maimour et al. [60], 2010		✓			✓	Cluster oriented routing in WSNs	3	Md
Bhattacharyya et al. [61], 2010	✓	✓	✓				3	Md
Singh et al. [62], 2010		✓			✓		2	Lw
Baranidharan et al. [63], 2010			✓				1	Lw
Celik et al. [64], 2010		✓	✓			Swarm intelligence based routing techniques in WSNs	3	Md
Modirkhazeni et al. [65], 2010		✓	✓			Security matrix for comparing multipath routing protocols	3	Md
Cecilio et al. [66], 2010	✓	✓	✓		✓		4	Md
Singh et al. [42], 2010					✓		1	Lw
Roseline et al. [67], 2011		✓					1	Lw
Raghunandan et al. [68], 2011		✓	✓		✓		3	Md
Wei et al. [69], 2011		✓			✓		2	Lw
Xu et al. [70], 2011			✓		✓		2	Lw
Ayaz et al. [71], 2011		✓	✓		✓	Routing approaches in underwater WSNs	4	Md
Saleem et al. [41], 2011	✓	✓	✓	✓	✓	In-depth taxonomy for categorizing routing protocols in WSNs, General framework for devising swarm based routing protocols	6	Hg
Sharma et al. [72], 2011		✓	✓			Secure hierarchical routing protocols in WSNs	3	Md
Kumar et al. [73], 2012	✓	✓				Homogeneous and heterogeneous WSNs oriented taxonomy	3	Md
Liu et al. [74], 2012	✓	✓	✓		✓	In-depth categorization of clustering techniques	5	Hg
Naeimi et al. [75], 2012	✓	✓			✓	In-depth classification of clustering techniques, Issues relevant to various phases of devised taxonomy	4	Md
Radi et al. [76], 2012	✓	✓	✓		✓	Classification of multipath routing techniques, Phasesof devising multipath routing protocol	5	Hg
Sumathi et al. [77], 2012		✓	✓			QoS-based routing in WSNs	3	Md
Modirkhazeni et al. [78], 2012	✓	✓	✓				3	Md
Saranya et al. [79], 2012		✓	✓				2	Lw
K. et al. [80], 2012		✓	✓			Node/Link disjoint multipath routing protocols in WSNs	3	Md
Uthra et al. [81], 2012	✓	✓	✓		✓	Network congestion-handling based QoS routing in WSNs	5	Hg
Zungeru et al. [82], 2012	✓	✓	✓		✓	Classical and swarm-intelligence oriented routing in WSNs	5	Hg
Ehsan et al. [38], 2012	✓	✓	✓		✓	Energy-efficient and QoS-aware routing approaches in WMSNs	5	Hg
Rahman et al. [83], 2013		✓	✓		✓		3	Md
El-Semary et al. [84], 2013	✓	✓			✓		3	Md
Manap et al. [85], 2013	✓		✓				2	Lw
Tyagi et al. [86], 2013	✓	✓	✓		✓	In-depth analysis of LEACH-based clustered routing protocols	5	Hg
Sikander et al. [87], 2013	✓		✓		✓		3	Md
Pantazis et al. [39], 2013	✓	✓	✓			Taxonomy based on network structure, communication model, topology and reliable routing	4	Md
Abazeed et al. [88], 2013		✓	✓				2	Lw
Sha et al. [37], 2013		✓	✓				2	Lw
Fersi et al. [89], 2013	✓	✓	✓		✓	Distributed Hash Table (DHT) oriented routing and data-management in WSNs	5	Hg
Masdari et al. [90], 2013		✓	✓				2	Lw
Soni et al. [91], 2014		✓	✓			Location-based routing in WSNs	3	Md
Tunca et al. [43], 2014	✓	✓	✓		✓	Mobile sink based routing in WSNs	5	Hg
Yu et al. [44], 2014	✓	✓	✓		✓	Mobile sink based state-of-the-art routing techniques in WSNs	5	Hg
Guo et al. [92], 2014	✓		✓			Intelligent energy-efficient routing in WSNs	3	Md
Zin et al. [40], 2014	✓	✓	✓		✓	State-of-the-art secure routing techniques in WSNs	5	Hg
Shamsan Saleh et al. [93], 2014	✓	✓	✓		✓	Energy-aware non-swarm and swarm-intelligence based routing in WSNs	5	Hg
Sara et al. [94], 2014	✓	✓	✓		✓	Taxonomy of mobility based WSNs routing protocols	5	Hg
Sharma et al. [95], 2015	✓	✓	✓				3	Md
Zin et al. [96], 2015	✓	✓	✓			Categorize secure-multipath routing protocols based on defense against particular attack	4	Md
Singh et al. [97], 2015		✓	✓		✓		3	Md
Mehmood et al. [98], 2015	✓	✓	✓				3	Md
Liu et al. [99], 2015	✓	✓	✓		✓	Evaluation of atypical hierarchical routing approaches in WSNs	5	Hg
Kumar et al. [100], 2016	✓	✓				Classification of biologically inspired algorithms in computer networks	3	Md
Jadhav et al. [101], 2016			✓		✓	Opportunistic routing protocols in WSNs	3	Md
Li et al. [102], 2016		✓	✓		✓	Intelligent cross-layered Underwater Acoustic Sensor Networks (UASNs) routing protocols, Expansion tendency of UASN routing protocols	4	Md
Gui et al. [103], 2016		✓	✓		✓	Discuss latest swarm oriented routing approaches and briefly portray a Spider Monkey Optimization (SMO) oriented routing protocol	4	Md
Singh et al. [104], 2016		✓	✓				2	Lw
Yan et al. [105], 2016	✓		✓		✓	Energy-efficient homogeneous and heterogeneous routing protocols with static and mobile topology	4	Md
Han et al. [106], 2016		✓	✓		✓	Green routing protocols for WMSNs	4	Md
Anisi et al. [107], 2017			✓		✓	Evaluation of energy harvesting methods and recent battery-oriented energy-efficient routing approaches	3	Md
Ahmed et al. [108], 2017	✓	✓	✓		✓	Evaluation of data forwarding routing protocols for Underwater Wireless Sensor Networks (UWSNs) using analytical/numerical simulation methods	5	Hg
Sabor et al. [109], 2017	✓		✓		✓	Latest mobility-oriented hierarchical routing protocols for WSNs	4	Md
Khalid et al. [110], 2017		✓	✓			Localization-oriented and localization-free routing protocols for UWSNs	3	Md
Hasan et al. [111], 2017	✓	✓	✓		✓	Multipath routing approaches with QoS guarantee in real-time WMSNs	5	Hg
Nayyar et al. [112], 2017					✓		1	Lw
Asif et al. [113], 2017		✓	✓		✓	QoS-aware routing approaches for WSNs, Up-to-date distribution of QoS literature and QoS parameters	4	Md
Rehan et al. [114], 2017	✓	✓	✓		✓	Evaluation and comparison of JOINT/DISJOINT single/multi-path and single/multi-radio multichannel routing protocols in WSNs	5	Hg

CNI* = Count Number Index, Rk* = Rank based on CNI, Hg* = High CNI with count 6 & 5, Md* = Medium CNI with count 4 & 3 and Lw* = Low CNI with count 2 & 1.

**Table 3:** Design analysis of surveys (with partial emphasis on routing in WSNs) under GSDF.

Surveys	FoA	DRF	PCA	CAN	FDT	NoR	CNI	Rk
Akyildiz et al. [115], 2002	✓	✓			✓	Communication layer-wise taxonomy of WSNs protocols	4	Md
Perrig et al. [116], 2004	✓	✓			✓	Security issues & challenges in WSNs	4	Md
Chen et al. [117], 2004	✓	✓			✓	Data-delivery model based QoS requirements	4	Md
Djenouri et al. [118], 2005	✓	✓			✓	Security problems at various network layers and solutions	4	Md
Wang et al. [119], 2006	✓	✓	✓		✓	Attacks at physical, data link, network, transport layers with possible solutions, Novel security taxonomy	5	Hg
Ren et al. [120], 2006	✓					Investigation of biologically inspired protocols for WSNs	2	Lw
Akyildiz et al. [121], 2007	✓	✓			✓	Communication layer-wise taxonomy of protocols for WMSNs	4	Md
Walters et al. [122], 2007	✓	✓					2	Lw
Li et al. [123], 2007		✓	✓		✓	QoS in real-time protocols for WSNs	4	Md
Akyildiz et al. [124], 2007	✓	✓			✓	State-of-the-art research in WMSNs	4	Md
Martinez et al. [125], 2007	✓		✓			Case-study to enhance learning based on real-world forest fire detection	3	Md
Yick et al. [126], 2008	✓	✓	✓		✓	Sensors internal platform, network services & protocol stack	5	Hg
Zhou et al. [127], 2008	✓	✓	✓		✓	Security issue and latest solutions, Identification of malicious threats affecting network operations	5	Hg
Anastasi et al. [128], 2009	✓	✓			✓	Taxonomy of energy conservation techniques	4	Md
Rehana et al. [129], 2009		✓					1	Lw
Chen et al. [130], 2009	✓	✓			✓	Hazards for WSNs & routing-layer based defense approaches	4	Md
Sen et al. [131], 2010		✓	✓		✓		3	Md
Halawani et al. [132], 2010		✓			✓	Network/MAC lifetime enhancement approaches in WSNs	3	Md
Bin et al. [133], 2011		✓	✓			Three rules based metric for designing new protocol, Energy conservation, routing and coverage in WSNs	3	Md
Kulkarni et al. [134], 2011	✓	✓	✓		✓	Computational Intelligence in WSNs	5	Hg
Saxena et al. [135], 2011	✓	✓					2	Lw
Soua et al. [136], 2011		✓				Classification of energy-efficient approaches in WSNs	2	Lw
Francesco et al. [137], 2011	✓	✓	✓		✓	Taxonomy of mobility based WSNs	5	Hg
Sen et al. [138], 2012	✓	✓	✓		✓		4	Md
Rault et al. [139], 2014	✓	✓	✓		✓	Trade-off vis-a-vis application demands and energy efficiency	5	Hg
Sergiou et al. [140], 2014	✓	✓			✓	Congestion control in WSNs, Guidelines for designing a new congestion control protocol in WSNs	4	Md
Kafi et al. [141], 2014	✓	✓	✓		✓	Congestion detection and control in WSNs	5	Hg
Bouaziz et al. [142], 2016	✓	✓	✓		✓	Mobility management in wireless sensor networks	5	Hg
Yadav et al. [143], 2016		✓	✓		✓	Energy-efficient data aggregation, clustering and routing protocols in WSNs	4	Md
Zenia et al. [144], 2016		✓	✓		✓	Evaluation of UWSNs based MAC & Routing protocols using comparison and simulation mechanisms	4	Md

CNI* = Count Number Index, Rk* = Rank based on CNI, Hg* = High CNI with count 6 & 5, Md* = Medium CNI with count 4 & 3 and Lw* = Low CNI with count 2 & 1.

**Table 4:** Design Analysis of Hierarchical Routing Surveys in WSNs.

Survey Type	Categorization and Design Requirements Based Analysis of Hierarchical Routing Surveys	
**Typical based**	*Hg*	[74,86]
	*Md*	[56,60,73,75]
	*Lw*	[69,70,85]
**Atypical based**	*Hg*	[99]
	*Md*	[87,97]
**Security based**	*Md*	[72,78]
**Energy Efficient**	*Md*	[98]
	*Lw*	[42,50]
**Mobile based**	*Md*	[109]

Hg* = High Count, Md* = Medium Count and Lw* = Low Count. Note: Various categories of count correspond to Count Number Index (CNI) in Table 2.

**Table 5:** Design analysis of classical routing surveys in WSNs.

Survey Type	Categorization and Design Requirements Based Analysis of Classical Routing Surveys	
**Structure based**	*Hg*	[35]
	*Md*	[36,46,47,52,53,54,61,66,68]
	*Lw*	[51,62,79,88,104]
**Operation based**	*Hg*	[35]
	*Md*	[36,46,47,52,53,61,66]
	*Lw*	[62,88]
**Partial based**	*Hg*	[126]
	*Md*	[115,121,124]

Hg* = High Count, Md* = Medium Count and Lw* = Low Count. Note: Various categories of count correspond to Count Number Index (CNI) in Table 2 and Table 3.

**Table 6:** Design analysis of energy-efficient routing surveys in WSNs.

Survey Type	Categorization and Design Requirements Based Analysis of Energy-efficient Routing Surveys	
**Intelligence based**	*Hg*	[93]
**Structure based**	*Md*	[39,83,95,98,105,107,132]
	*Lw*	[42,50,63,67]
**Operation based**	*Hg*	[38]
	*Md*	[39,95,105,106,107]
**Partial based**	*Hg*	[139]
	*Md*	[128,133,143]
	*Lw*	[135,136]

Hg* = High Count, Md* = Medium Count and Lw* = Low Count. Note: Various categories of count correspond to Count Number Index (CNI) in Table 2 and Table 3.

**Table 7:** Design analysis of secure routing surveys in WSNs.

Survey Type	Categorization and Design Requirements Based Analysis of Secure Routing Surveys	
**Multipath based**	*Hg*	[59]
	*Md*	[65,96]
**Hierarchical based**	*Md*	[72,78]
**Typical based**	*Hg*	[40]
	*Md*	[84]
	*Lw*	[45]
**Partial based**	*Hg*	[119,127]
	*Md*	[116,118,130,131,138]
	*Lw*	[122,129]

Hg* = High Count, Md* = Medium Count and Lw* = Low Count. Note: Various categories of count correspond to Count Number Index (CNI) in Table 2 and Table 3.

**Table 8:** Design analysis of multipath-based routing surveys in WSNs.

Survey Type	Categorization and Design Requirements Based Analysis of Multipath-based Routing Surveys	
**Typical based**	*Hg*	[76,111]
	*Md*	[80]
	*Lw*	[37,90]
**Multichannel Routing**	*Hg*	[114]
**Security based**	*Hg*	[59]
	*Md*	[65,96]
**Fault-Tolerant**	*Md*	[58]
	*Lw*	[90]

Hg* = High Count, Md* = Medium Count and Lw* = Low Count. Note: Various categories of count correspond to Count Number Index (CNI) in Table 2.

**Table 9:** Design analysis of computationally-intelligent routing surveys in WSNs.

Survey Type	Categorization and Design Requirements Based Analysis of Computationally-Intelligent Routing Surveys	
**Swarm based**	*Hg*	[41]
	*Md*	[64,100,103]
	*Lw*	[112]
**Hybrid based**	*Md*	[48,92]
**Mixed**	*Hg*	[82,93]
**Partial based**	*Hg*	[134]
	*Lw*	[120]

Hg* = High Count, Md* = Medium Count and Lw* = Low Count. Note: Various categories of count correspond to Count Number Index (CNI) in Table 2 and Table 3.

**Table 10:** Design analysis of QoS-based routing surveys in WSNs.

Survey Type	Categorization and Design Requirements Based Analysis of QoS-based Routing Surveys	
**Typical based**	*Md*	[77,106,113]
	*Lw*	[50]
**Multipath based**	*Hg*	[111]
**Fault-Tolerance based**	*Md*	[58]
**Cong.-Handling based**	*Hg*	[81]
**Partial based**	*Hg*	[141]
	*Md*	[117,123,125,140]

Hg* = High Count, Md* = Medium Count and Lw* = Low Count. Note: Various categories of count correspond to Count Number Index (CNI) in Table 2 and Table 3.

**Table 11:** Design analysis of geographic routing surveys in WSNs.

Survey Type	Categorization and Design Requirements Based Analysis of Geographic Routing Surveys	
**Mobile**	*Hg*	[43,44,94]
	*Md*	[109]
**Location based**	*Md*	[91]
	*Lw*	[55]
**Partial based**	*Hg*	[137,142]

Hg* = High Count, Md* = Medium Count and Lw* = Low Count. Note: Various categories of count correspond to Count Number Index (CNI) in Table 2 and Table 3.

**Table 12:** Design analysis of other least-focused areas of routing surveys publication in WSNs.

Survey Type	Categorization and Design Requirements Based Analysis of Other Least-Focused Areas of Routing Surveys	
**In-network Fusion**	*Md*	[49]
**DHT based**	*Hg*	[89]
	*Md*	[57]
**UWSNs based**	*Hg*	[108]
	*Md*	[71,102,110]
(Partial) — >	*Md*	[144]
**Opportunistic based**	*Md*	[101]
**Multichannel Routing**	*Hg*	[114]

Hg* = High Count, Md* = Medium Count and Lw* = Low Count. Note: Various categories of count correspond to Count Number Index (CNI) in Table 2 and Table 3.
